# Impact of metallic nanoparticles on gut microbiota modulation in colorectal cancer: A review

**DOI:** 10.1002/cai2.150

**Published:** 2024-10-11

**Authors:** Akash Kumar, Jhilam Pramanik, Kajol Batta, Pooja Bamal, Mukesh Gaur, Sarvesh Rustagi, Bhupendra G. Prajapati, Sankha Bhattacharya

**Affiliations:** ^1^ Department of Food Technology SRM University, Delhi NCR Sonepat India; ^2^ MMICT & BM (Hotel Management), Maharishi Markandeshwar (Deemed to be University) Mullana India; ^3^ Department of Food Technology William Carey University Shillong India; ^4^ Department of Food Technology ITM University Gwalior India; ^5^ Department of Food Technology Chaudhary Devi Lal University Sirsa India; ^6^ Department of Food Technology Guru Jambheshwar University of Science and Technology Hisar India; ^7^ School of Applied and Life Sciences Uttaranchal University Dehradun India; ^8^ Shree S. K. Patel College of Pharmaceutical Education and Research Ganpat University Mehsana India; ^9^ Department of Pharmaceutics School of Pharmacy & Technology Management, SVKM'S NMIMS Deemed‐to‐be University Shirpur Maharashtra India

**Keywords:** apoptosis, carcinogenesis, dysbiosis, gastrointestinal microbiome, titanium dioxide

## Abstract

Colorectal cancer (CRC) is the third most prevalent cancer. Ongoing research aims to uncover the causes of CRC, with a growing focus on the role of gut microbiota (GM) in carcinogenesis. The GM influences CRC development, progression, treatment efficacy, and therapeutic toxicities. For example, *Fusobacterium nucleatum* and *Escherichia coli* can regulate microbial gene expression through the incorporation of human small noncode RNA and potentially contribute to cancer progression. Metallic nanoparticles (MNPs) have both negative and positive impacts on GM, depending on their type. Several studies state that titanium dioxide may increase the diversity, richness, and abundance of probiotics bacteria, whereas other studies demonstrate dose‐dependent GM dysbiosis. The MNPs offer cytotoxicity through the modulation of MAPK signaling pathways, NF‐kB signaling pathways, PI3K/Akt signaling pathways, extrinsic signaling pathways, intrinsic apoptosis, and cell cycle arrest at G1, G2, or M phase. MNPs enhance drug delivery, enable targeted therapy, and may restore GM. However, there is a need to conduct well‐designed clinical trials to assess the toxicity, safety, and effectiveness of MNPs‐based CRC therapies.

## Graphical abstract

Metallic nanoparticles have the potential to be a new and effective treatment for colorectal cancer.

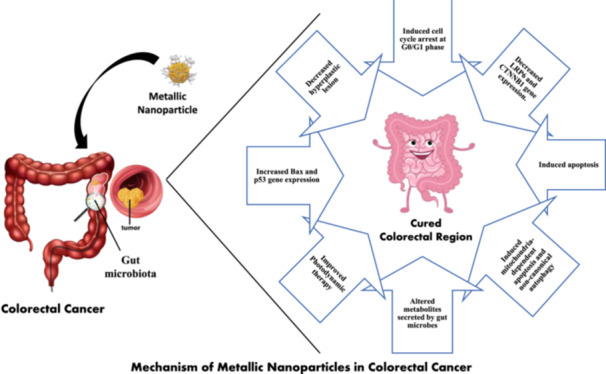

**Abbreviations**
5‐FU: 5‐fluorouracil
ACP NP: amorphous calcium phosphate nanoparticle
AgNP: silver nanoparticle
AuNP: gold nanoparticle
CA 19‐9: cancer antigen 19‐9
CEA: carcinoembryonic antigen
CRC: colorectal cancer
CuO NPs: copper oxide nanoparticle
CVD: cardiovascular disease
DNA: deoxyribonucleic acid
Fe_3_O_4_ NP: iron oxide nanoparticle
GIT: gastrointestinal tract
GM: gut microbiota
HB: honeybush
IC_50_
: half‐maximal inhibitory concentration
LRP6: low‐density lipoprotein receptor‐related protein 6 gene
MAPK: mitogen‐activated protein kinases
MnNP: manganese nanoparticle
MNP: metallic nanoparticle
Mo‐ZnO/RGO NC: molybdenum‐zinc oxide/reduced graphene oxide nanocomposite
NA: not available
Nd:YAG: neodymium‐doped yttrium aluminum garnet
NF‐kB: nuclear factor kappa B
NP: nanoparticle
PI3K/Akt: phosphoinositide 3‐kinases/Protein kinase B
Pt NP: platinum nanoparticle
RNA: ribonucleic acid
ROS: reactive oxygen species
SCFA: short‐chain fatty acid
TiO_2_ NP: titanium dioxide nanoparticle
Ti:Sapphire: titanium–sapphire
ZnO NP: zinc oxide nanoparticle

## 1 INTRODUCTION

**Figure 1:**
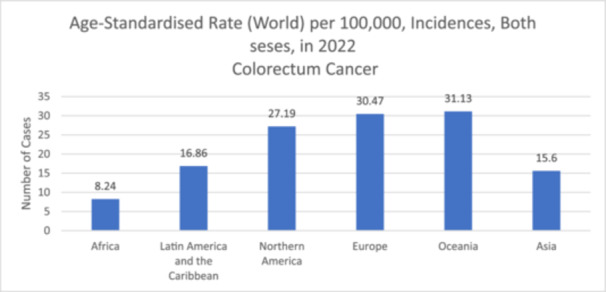
Age‐standardized rate for colorectum cancer worldwide in 2022, all ages and both genders. *Data source*: GLOBOCAN 2022 (version 1.1); IARC (https://gco.iarc.who.int) World Health Organization [5].

In the gastrointestinal tract (GIT), various communities of microbes reside, known as gut microbiota (GM), and play a crucial role in maintaining overall health and well‐being [1, 2]. Gut dysbiosis is linked with the occurrence of various diseases, including colorectal cancer (CRC) [3]. CRC is the third most prevalent cancer (1.9 million newly diagnosed cases annually) and ranked as the second cause of cancer mortalities (0.9 million deaths annually) [4]. It is estimated that the burden of this disease will increase to 1.6 million deaths by 2040. Therefore, early diagnosis is important to increase the survival rates [4]. General risk factors for CRC include age (higher risk over 50), family history or genetic conditions, personal history of tumor or polyps, sedentary behavior, poor diet, obesity, smoking, and excessive alcohol consumption [4]. Figure 1 represents the 2022 global age‐standardized rate for colorectum cancer across all ages and genders [5].

Ongoing research aims to uncover the causes of CRC, with a growing focus on the role of GM in carcinogenesis [6]. The large population of bacteria resides in the host gut in a mutually beneficial relationship, but alterations in the gut environment can trigger the growth of pathogenic microbes [7]. The GM influences CRC development, progression, treatment efficacy, and therapeutic toxicities [8, 9]. Technologies like 16S rRNA sequencing have advanced our understanding of the gut microbiome's impact on human health [10]. Gut microbial metabolites like polyamines, tryptophan catabolites, vitamins, polyphenols, and short‐chain fatty acids (SCFAs) can affect the formation, progression, and metastasis of CRC [11, 12]. *Bacteroidetes* and *Firmicutes* are more abundant in patients with CRC; however, bacterial diversity and abundance are less as compared to healthy individuals [13]. *Streptococcus gallolyticus, Enterococcus faecalis, Fusobacterium nucleatum*, and *Escherichia coli* are associated with the onset and progression of CRC. Studies have suggested that *F. nucleatum* abundance is a potential prognostic biomarker in metastatic CRC (mCRC) [14]. The administration of *S. gallolyticus* resulted in a significant increase in several key tumorigenic parameters compared to control mice receiving nontumorigenic bacteria. These parameters include increased tumor formation, elevated tumor burden, enhanced dysplasia grade, stimulated cell proliferation, and upregulated β‐catenin staining [15].

Exposure to metallic nanoparticles (MNPs) can modulate the GM composition [16, 17, 18, 19], and our body is daily exposed to MNPs. For example, silver is used as a coloring substance in the food industry and its exposure levels range from 0.03 to 2.6 μg/kg bw/d [20]. Similarly, titanium dioxide (TiO_2_) (E171) is used in confectionery foods as a brightening agent [21, 22, 23], and silicon dioxide (SiO_2_) (E551) is used as an anticaking agent in powdered food products [24, 25]. The EFSA estimated that daily exposure to E551 is highest in infants (0.8 and 74.2 mg/kg bw/d), followed by children and adults [26]. Various animal studies have demonstrated that the administration of MNPs increased microbial species richness and diversity. These studies also highlighted that the abundance of the genus *Lactobacillus* and *Bifidobacterium* was also increased [19, 27, 28, 29, 30, 31]. A study showed that exposure to TiO_2_ NPs increased the abundance of *Lactobacillus reuteri* [19]. In another study, chickens were fed varying doses of selenium nanoparticles (Se NPs) (0, 0.3, 0.9, and 1.5 mg/kg in their feed). The results showed that Se NP led to an increase in the abundance of Lactobacillus and Faecalibacterium. Additionally, the production of butyric acid was also increased [28]. However, some studies have shown negative impacts on GM composition [32]. In a study, Cu NPs or Ag NPs (500 mg/kg food) were fed to zebrafish for 14 days. The results showed that Cu NPs reduced the abundance of beneficial bacteria. Specifically, *Cetobacterium somerae* up to a nondetectable level [33]. In another study, the diet of zebrafish was supplemented with TiO_2_ NPs along with bisphenol A, which resulted in an increased abundance of pathogenic bacteria such as *Lawsonia* and a reduced abundance of beneficial bacteria such as *Hyphomicrobium* [34].

This highlights that MNPs can play an important role in CRC by modulating GM.

Additionally, MNPs can be used for diagnostic and therapeutic purposes of CRC [35]. For example, iron oxide (Fe_3_O_4_) NPs have shown diverse biological properties, including their ability to enhance the effectiveness of anticancer drugs like 5‐fluorouracil (5‐FU) through magnetic hyperthermia [36]. Fe_3_O_4_ NPs containing 5‐FU were suitable for use in combined thermo‐chemotherapeutic therapy when paired with magnetic hyperthermia. The use of MNPs caused a noticeable tumor regression. Compared to magnetic hyperthermia or 5‐FU alone, the thermo‐chemotherapeutic tumor treatment has proven to be significantly better [36]. They have also been used to efficiently deliver epirubicin to colon carcinoma cells, allowing for tumor detection by magnetic resonance imaging (MRI) [37]. Additionally, MNPs such as gold and silver show immunomodulatory properties [35, 38, 39] that may uplift the lifespan of CRC patients [39]. With nanomedicine advancements, MNPs are used in various ways in different industries, so it is important to understand the impact of MNPs on GM and CRC. This review explores the role of MNPs in CRC development, progression, and therapy by modulating the GM. Additionally, we emphasize the direct impact of MNPs on CRC.

## 2 GUT MICROBIOME DYSBIOSIS IN COLON CANCER

**Figure 2:**
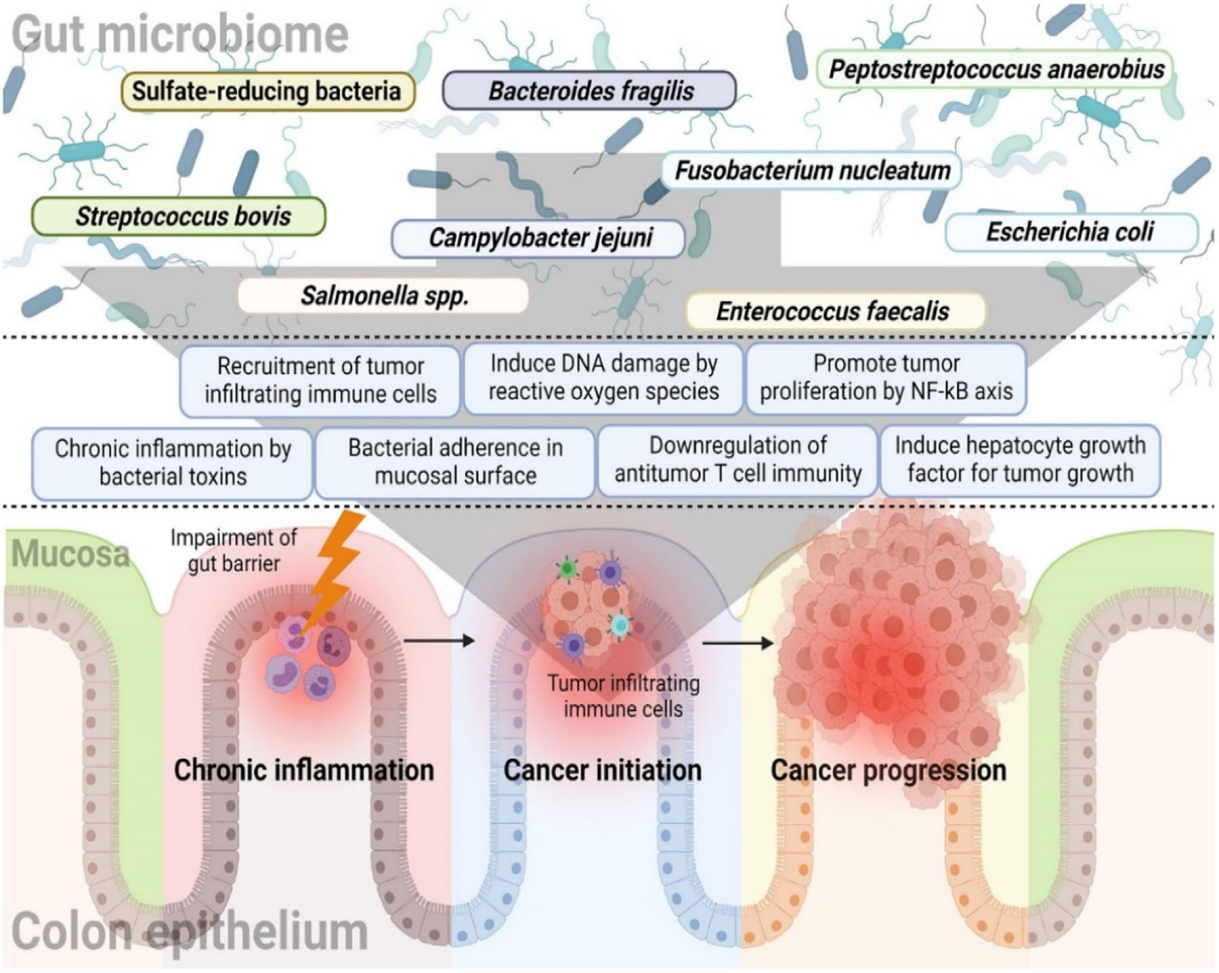
The relationship between the gut microbiome and colorectal carcinoma. Figure is reproduced from Kim and Lee [12] under the CC‐BY license.

As previously mentioned, gut dysbiosis is one of the main factors influencing CRC development. Figure 2 represents the relationship between GM and CRC [12]. The GM offers various functions, including the digestion, regulation of the immune response, and synthesis of essential nutrients [40]. The maintenance of GMs is important for an individual's overall health [1]. Commensal bacteria in the intestine stimulate innate immunity by regulating toll‐like receptors, activating T‐cells, and generating antimicrobial peptides through the nuclear factor‐kappa B signaling pathway [41].

Furthermore, the microbes inhabiting the GIT engage in complex interactions with each other and with the host. This communication network can have profound effects on both disease development and overall health [42]. This interaction extends to the realm of carcinogenesis, where the gut microbiome's role becomes particularly significant [6]. Certain bacteria have been observed to proliferate in CRC patients, whereas others decline. Bacteria such as *Fusobacteria, Methanobacteriales, Coriobacteridae, Akkermansia* spp., *Porphyromonadaceae,* and *Alistipes* tend to increase in abundance in GIT of CRC patients [43, 44].

Studies have revealed that specific bacteria like *F. nucleatum* and *E. coli* can regulate microbial gene expression by incorporating human small noncode RNA and potentially contribute to cancer progression [45]. Gram‐negative bacteria such as *Parabacteroides*, *Akkermansia, Bacteroides,* and *Alistipes* have shown positive associations with tumor development, whereas Gram‐positive bacteria like *Clostridium* group XIVa show negative associations with tumor generation. Bacteria such as *Roseburia, Treponema*, *Faecalibacterium* spp., *Ruminococcus, Lactobacillus,* and *Bifidobacterium* tend to decrease in abundance in CRC patients [43, 44]. There is a growing demand to explore the impact of GM in the prevention and management of CRC. This could involve dietary interventions, probiotics, prebiotics, or even more advanced microbiome‐based therapies.

## 3 MNPs: PROPERTIES AND APPLICATIONS

MNPs have emerged at the forefront of research and innovation in the fields of nanotechnology and materials science. Noble metals such as platinum, zinc, silver, gold, copper, and others show unique and remarkable properties that make them exceptionally attractive for various applications [46]. MNPs exhibit surface plasmon resonance when their size lies in the size range of 10–100 nm. This phenomenon occurs due to the collective oscillation of conduction electrons on the MNP surface, which becomes resonant with the incident light's wavelength. The interaction leads to strong light absorption and scattering, resulting in unique optical properties [47, 48]. A key factor driving the application of MNPs in the pharmaceutical industry is their high surface‐to‐volume ratio. This translates to a vast surface area relative to their volume, allowing for significant interaction with surrounding molecules. This characteristic makes MNPs potential candidates for a process called functionalization, where their surface is strategically modified with desired biomolecules [49].

The MNPs have vast applications in diverse fields, including disease diagnosis and treatment, catalysis, composite materials preparation, labeling of optoelectronic recorded media, and sensor technology [50]. For example, gold nanoparticles are employed extensively in medicines, drug delivery, and diagnostics [51, 52]. Silver nanoparticles (AgNPs) are renowned for their antimicrobial and anti‐inflammatory properties, making them crucial in wound healing, pharmaceutical formulations, and medical implant coatings [53, 54]. MNPs can be used as biosensors. Elshafey et al. employed gold nanoparticles (AuNPs) conjugated with protein G to develop a sensitive immunosensor for epidermal growth factor receptor detection. This sensor achieved a remarkable limit of detection of 0.88 pg/mL in human plasma [55].

## 4 RELATIONSHIP BETWEEN MNPs, GUT MICROBIOME, AND COLON CANCER

Daily people are exposed to MNPs as these may be used as additives and packaging material in food industries. However, depending on MNP types and sources, they can interact and modulate the GM [56]. Nanoparticles alter the various physiological functions, such as neurobehavioral, metabolism, and immunity, through the modulation of GM [57, 58]. They can directly affect the composition and activities of gut microflora [59]. The interplay between nanomaterials (NMs) and GM is a multifaceted, two‐way relationship. NMs can impact both the structure and function of GM. The composition and activity of GM can influence the behavior and toxicity of NMs [60]. Disruptions in the GM caused by certain nanoparticles (e.g., Gold [Au] NPs, Silver [Ag] NPs, and Titanium [Ti] Dioxide NPs) can have significant health consequences. These disruptions, influenced by nanoparticle properties like size, shape, composition, and surface chemistry, lead to decreased microbial diversity and beneficial bacteria in the gut. This disturbance can trigger inflammatory responses locally and throughout the body, affecting various physiological systems [61]. Certain inorganic NPs, such as silver, titanium dioxide, silicon dioxide, and zinc oxide, can alter GM composition the composition by interacting with the immune system. This alteration gives rise to various chronic diseases, including inflammatory bowel disease (IBD), diabetes, and even CRC [62, 63].

AgNPs are generally used for their antimicrobial properties in many products but may alter the GM and make the host more susceptible to various diseases [64]. However, the mucus lining in the gut prevents the absorption of AgNPs into intestinal cells, limiting their impact on gut microflora. Wang et al. conducted an animal study in which AgNPs were administrated to mice and the results showed a significant decline in the abundance of Firmicutes and an incline in the abundance of Bacteroidetes [65]. As stated above the decline in Firmicutes provides conflicting insights [66, 67], therefore it is difficult to correlate this with CRC. In a study, zebrafish were exposed to abalone viscera hydrolysates decorated AgNPs and the results showed a significant reduction in *Flavobacterium, Bacteroidetes*, *Halomonas*, *Pseudomonas alcaligenes*, and *Plesimonas shigelloides*. However, the abundance of *Actinobacteria*, *Burkholderia, Caballeronia, Paraburkholderia, Proteobacteria, Sphingomonas,* and *Rhodococcus* increased significantly [32]. A study highlighted that *Bacteroidetes* are higher in CRC patients [68], specifically, *F. nucleatum (*a species belonging to *Bacteroidetes)* [69].

In a study, Ag NPs of varying sizes (10, 20, 40, 60, and 100 nm) were used to evaluate their cytotoxicity on LoVo cells. The findings revealed a size‐dependent cytotoxic effect. Smaller Ag NPs showed greater cytotoxicity than larger NPs. This may occur due to the enhanced cellular uptake of smaller NPs, as they can penetrate the cell membrane easily [70]. Williams et al. reported that small‐size Ag NPs increased intestinal permeability upon exposure. This increased permeability elevated intestinal inflammation and enhanced the translocation of antigens and microorganisms into the circulatory system [71]. Long‐term oral exposure to Ag NPs may damage the epithelial structure, reduce the mucosal layer's thickness, and alter the gut flora. Additionally, this study revealed that long‐term exposure resulted in reactive oxygen species (ROS) formation and uncontrolled apoptosis [72].

A study reported that titanium dioxide NPs can result in undesirable GM alterations. The administration of titanium dioxide NPs may impact the balance between commensal and pathogenic bacteria, resulting in oxidative stress and inflammatory reactions [18], which are one of the factors of initiation, development, and progression of CRC. Chronic oxidative stress can oxidize the biomolecules or activate the inflammatory signaling pathways, triggering the expression of transcription factors and dysregulating the gene and protein expression which can contribute to tumor initiation [73].

In a study, zinc nanoparticles (produced via *Bacillus subtilis*) were fed to broilers, and results demonstrated a reduction in Coliform, *E. coli*, and salmonella [74]. Some studies have highlighted that an increased abundance of *E. coli* could be a cofactor in the pathogenesis of CRC [75]. The administration of ZnO NP at a dosage of 20 μg/mL to children suffering from autism spectrum disorder resulted in the increased abundance of *Actinobacteria* and *Firmicutes* from 14.26% to 40.4% and from 27.9% to 47.7%, respectively. However, the abundance of *Proteobacteria* decreased from 57.6% to 8.8% [76]. The human study found an increased abundance of *Firmicutes* and a decreased abundance of *Proteobacteria* in CRC patients [66]. However, a study highlighted that the abundance of *Firmicutes* phylum is reduced in CRC patients [67]. These conflicting results highlight the complexity of the gut microbiome and its relationship to CRC. Therefore, detailed research is necessary to understand the impact of zinc nanoparticles on CRC development, progression, or treatment by modulating GM.

A study demonstrated an increased abundance of *Bifidobacterium* and *Lactobacillus* after exposure to TiO_2_ + Zn and SiO_2_ + Zn. However, TiO_2_ + Fe, SiO_2_ + Fe, and water + Zn significantly reduced the *Bifidobacterium* and *Lactobacillus.* The study suggested that combining TiO_2_ and SiO_2_ with Zn may promote the growth of probiotic bacteria in the gut of broilers [77], which may reduce the risk of CRC [78]. The reduced abundance of probiotics may result in the development and progression of CRC [79].

A study investigated the effects of Ag, SiO_2_, and TiO_2_ (at varying doses from 0 to 400 mg/kg) nanoparticles on the GM of C57BL/6JRj mice. The results showed that Ag NPs exposure reduced the abundance of *Bilophila* bacteria and TiO_2_ NPs increased the abundance of *Cyanobacteria* and decreased the abundance of *Tenericutes*. However, SiO_2_ NPs did not significantly impact bacterial groups [80]. There are higher levels of *Bilophila* in the GM of patients with CRC compared to healthy individuals [81].

All the above‐discussed studies have shown that MNPs modulate the gut microflora (shown in Table 1) and these modulations may have a positive or negative impact on CRC. Therefore, understanding these interactions is essential for assessing their impact on human health.

**Table 1:** Overview of modulation of gut microbiome under the influence of specific nanoparticles.

Nanoparticles	Model	Test concentration	Outcome	References
Zn	Autism Spectrum Disorder Children	20 μg/mL	↑ *Actinobacteria* and *Firmicutes* ↓ *Proteobacteria*	[76]
Ag	Mice	NA	↑ Firmicutes ↓ Bacteroidetes	[65]
Ag	Zebrafish	9 and 18 μg/L	↑ Rhodococcus erythropolis ↓ *Plesimonas shigelloides*, *Pseudomonas alcaligenes, Flavobacterium*	[32]
Silica	Sprague Dawley rats	50 mg/kg	↑ *Candidatus Saccharibacteria* ↓ *Verrucomicrobia*	[82]
Si	Balb/c Mice	40–80 mg/kg	↑ Lactobacillus, Sphingomonas, Sutterella, Akkermansia, and Prevotella ↓ Ruminococcus and Allobaculum	[83]
TiO_2_	Wistar rats	50 mg/kg	↑ *Lactobacillus reuteri* ↓ *Romboutsia*	[19]
Ag, SiO_2_, and TiO_2_	C57BL/6JRj mice	0–400 μg/kg	Dose‐dependent ↓ *Bilophila* (after exposure to Ag NP) Dose‐dependent ↑ Cyanobacteria and ↓ Tenericutes (after exposure to TiO_2_NP)	[80]
TiO_2_, SiO_2_, and ZnO	Broiler	9.7 × 10^−2^ mg NP/mL	↑ *Bifidobacterium* or *Lactobacillus* (in TiO_2_ + Zn and SiO_2_ + Zn) ↓ *Bifidobacterium* or *Lactobacillus* (in TiO_2_ + Fe, SiO_2_ + Fe, and water + Zn)	[77]
Zn	C57BL/6J mice	30 mg Zn/kg	*Allobaculum* was significantly higher in male mice compared to females, while the abundance of *AF12* was vice‐versa *Coriobacteriaceae* was found only in male mice	[84]
ZnO	Broilers	40 g/kg	↓ *Coliform, E. coli*, and *salmonella*	[74]

## 5 THERAGNOSTIC IMPACT OF MNPs ON CRC

**Figure 3:**
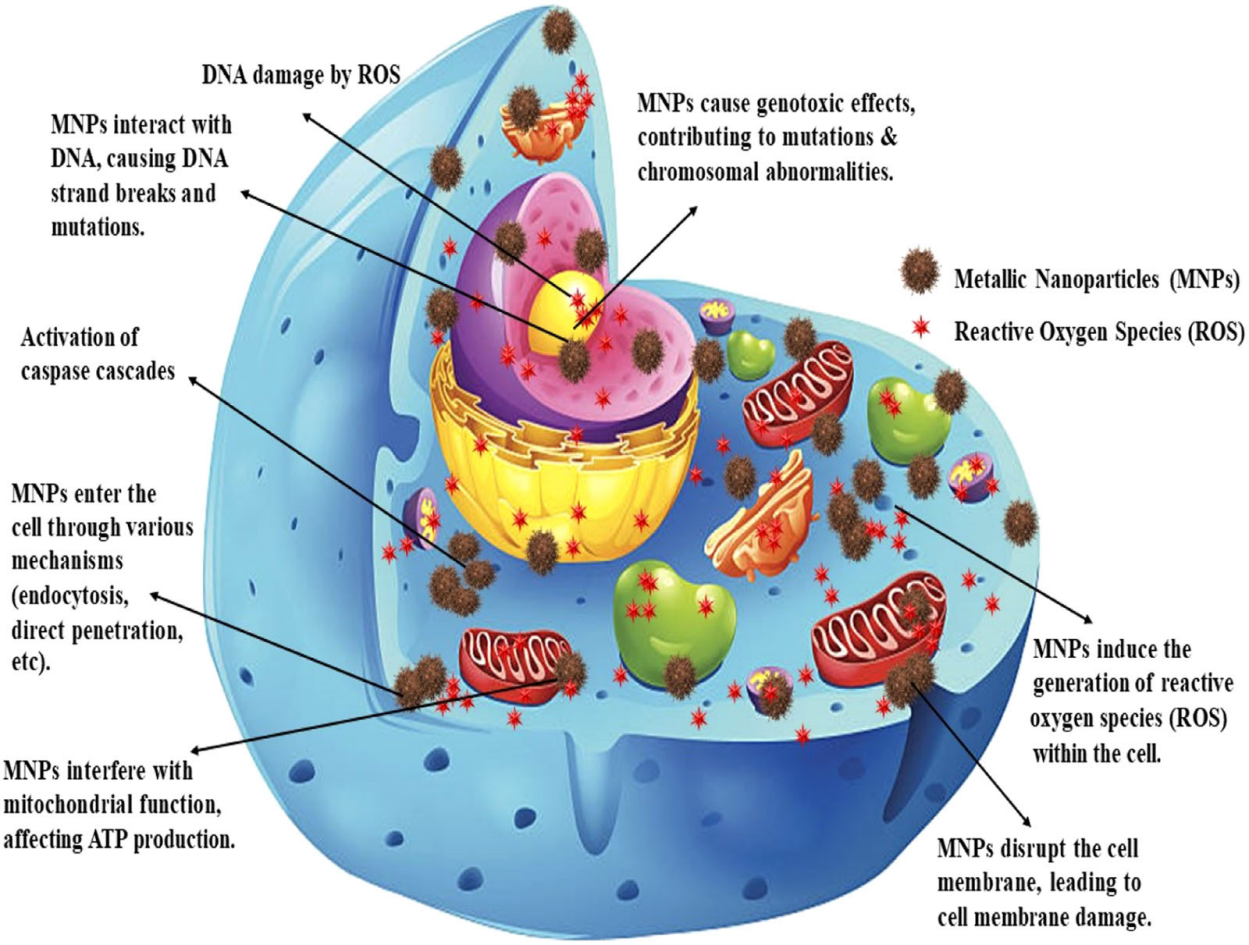
The mechanism of cytotoxicity induced by MNPs.

Various studies have explored the impact of different types of MNPs on colon cancer. Figure 3 illustrates the mechanism of cytotoxicity induced by MNPs. MNPs induce cytotoxicity through various mechanisms, potentially leading to cell death. MNPs can interact with the cell membrane, resulting in physical damage and compromising its integrity. This leads to the disruption in cellular homeostasis and essential functions [85]. Additionally, MNPs can directly interact with DNA and break down the strands. They also produce ROS that indirectly damage DNA through oxidative stress. Damaged DNA can lead to cell cycle arrest or mutations, finally leading to cell death [86, 87, 88]. MNPs trigger the activation of the caspase cascade, resulting in programmed cell death (apoptosis) [89].

The MNPs increased the abundance of the genus *Lactobacillus* and *Bifidobacterium* [19, 27, 28, 29, 30, 31], which produce SCFAs [90]. SCFAs play a crucial role in maintaining cell homeostasis by impacting histone deacetylases, chemotaxis, cytokine production, immune cell migration, cell attachment, and programmed cell death [91]. Modulation of SCFA levels can be used for colon cancer treatment and prevention [92]. The genus Enterococcus can contribute to the development of CRC by producing cancer‐causing microbial metabolites and secreting virulence factors that promote cancer growth [93], whereas selenium nanoparticles show antibacterial properties against *Enterococcus cecorum* and seem to be a potential approach to control poultry disease without significantly affecting the commensal population in the gut of the livestock [16].

Researchers have found that zinc oxide NPs (ZnO NPs), especially those with large aspect ratios, exhibit cytotoxicity. Additionally, ZnO NPs have been biosynthesized using egg albumin, resulting in significant cytotoxicity on cancer cells such as MCF‐7. The cytotoxic effect is attributed to an increase in ROS levels, which regulates the transcription of apoptotic genes and reduces the expression of antiapoptotic genes like B‐cell lymphoma‐2 [94, 95, 96].

**Figure 4:**
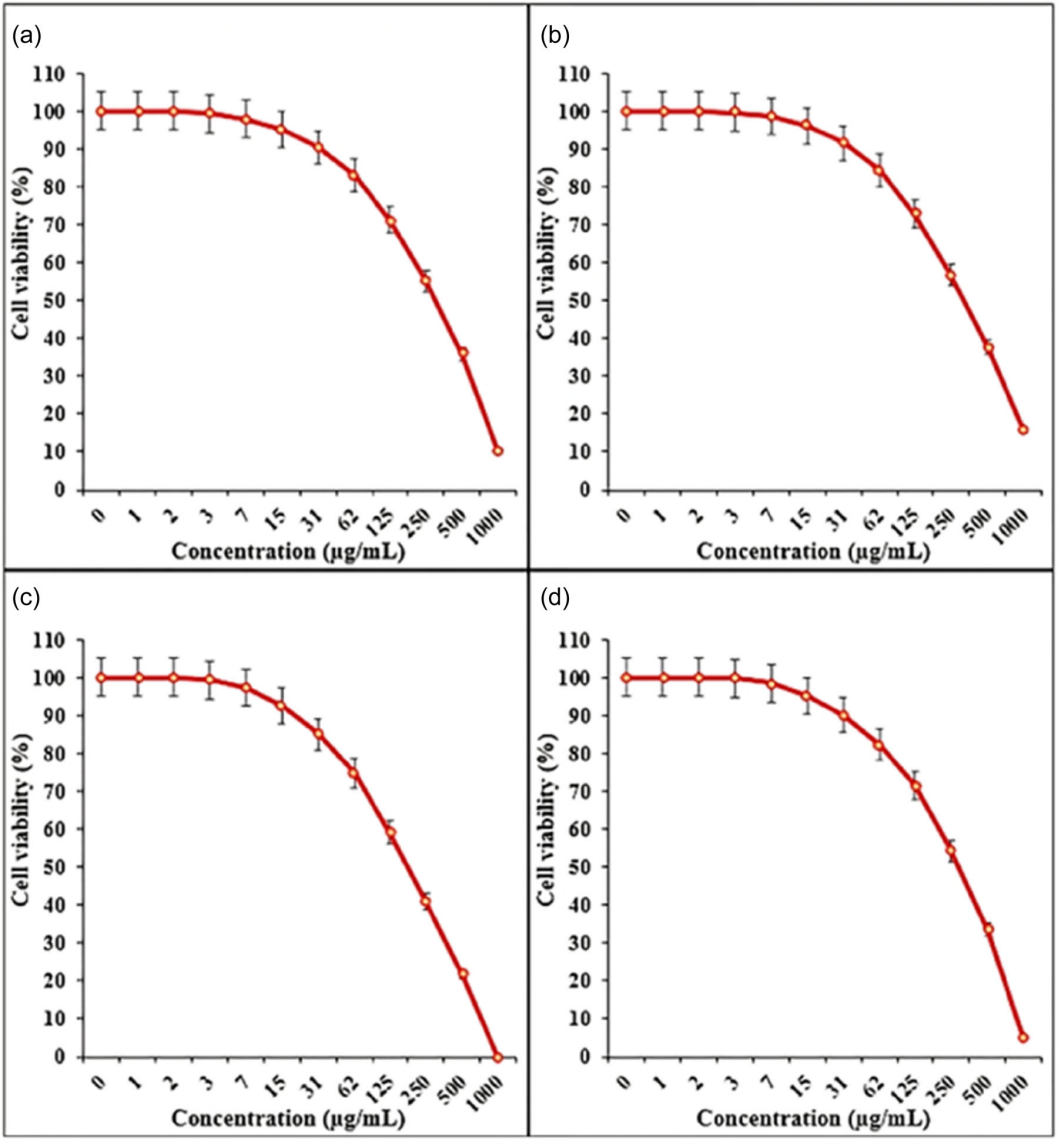
Impact of Fe_3_O_4_/Pectin NPs on cell viability in various cancer cell lines (a) Ramos.2G6.4C10, (b) HCT‐8 [HRT‐18], (c) HCT 116, and (d) HT‐29. NP, nanoparticle. Figure is reproduced from Wang et al. [97] under the CC‐BY license.

In an investigation, Fe_3_O_4_/Pectin NPs showed a dose‐dependently impact on the viability of CRC cell lines (Ramos.2G6.4C10 (I), HCT‐8 [HRT‐18] (II), HCT‐116 (III), and HT‐29 (IV)) (shown in Figure 4) with IC_50_ values of 317, 337, 187, and 300 µg/mL, respectively. This suggests their potential for selective cancer therapy [97]. Yusefi et al. developed spherical Fe_3_O_4_ NPs from *Garcinia mangostana* extract of 13.42 ± 1.58 nm mean diameter. The NPs demonstrated cytotoxicity against HCT‐116, with IC_50_ values of 99.80 µg/mL [98]. Chitosan‐coated Fe_3_O_4_ NPs enhanced ROS production and induced cell death through the activity of caspase 9/3 in colorectal carcinoma cells. The in vitro MRI studies in human embryonic kidney cells and HCT‐116 demonstrated that nanohybrid can be used as a contrast agent in MRI for CRC detection [89].

**Figure 5:**
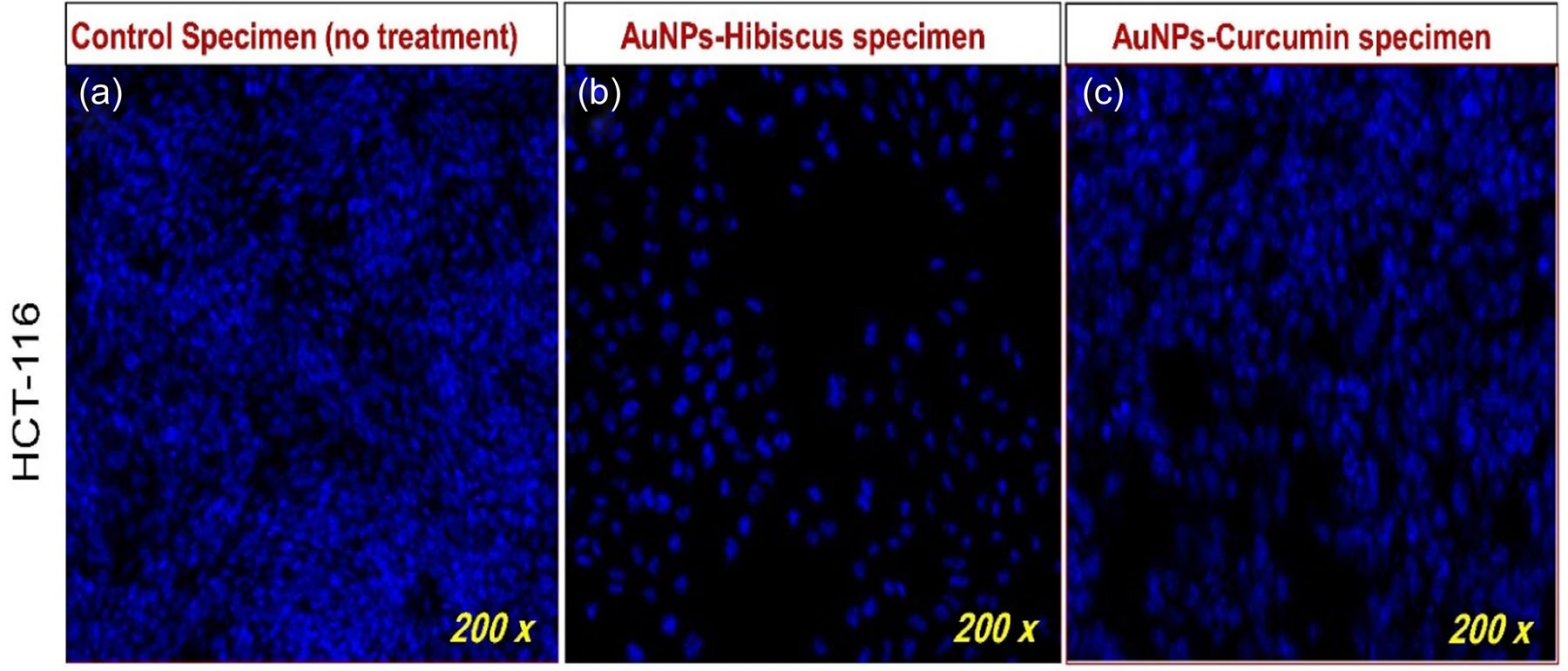
Disintegration of colon cancer cell DNA. (a–c) Impact of AuNPs‐Hibiscus and AuNPs‐Curcumin on colon cancer cells (HCT‐116) post 48 h treatment (0.8 µg/mL). AUNP, gold nanoparticle. Figure is reproduced from Akhtar et al. [102] under the CC‐BY license.

Akbarzadeh Khiavi et al. [99] developed RNase A functionalized AuNPs and stabilized them with polyethylene glycol (PEG). The developed MNP exhibited cytotoxicity against SW‐48. The results showed an increment in ROS levels, which may be responsible for apoptosis activity against SW‐48 [99]. AuNPs functionalized with cetuximab have a stronger cytotoxic effect than cetuximab alone against HT‐29 CRC cells. C‐AuNPs inhibited cell proliferation and induced apoptosis [100]. Additionally, C‐AuNPs as carriers of 5‐FU on CRC cells (HCT‐116 and HT‐29) induced apoptosis and necrosis and inhibited cell proliferation, suggesting enhanced chemotherapeutic effects [101]. The disintegration of colon cancer cells (HCT‐116) DNA after 48 h of treatment of AuNPs‐Hibiscus and AuNPs‐Curcumin at 8 µg/mL is shown in Figure 5 [102]. Gold NPs can be used for drug delivery and cancer targeting. They may enhance the delivery efficacy of drugs like cisplatin and have diagnostic applications as well [103].

AgNPs were synthesized using rhizomes from three different species of Curcuma. The nanoparticles significantly inhibited the growth of HT‐29 colon cancer cells, demonstrating their potential anticancer effects [104]. Ag‐NPs synthesized from *Perilla frutescens* leaves demonstrated significant anticancer activity with an IC_50_ value of 39.28 μg/mL in human colon carcinoma cell line COLO205 [105]. Similarly, Ag‐NPs derived from *Mimusops elengi* fruits inhibited colon cancer cells (HT‐29) in a dose‐dependent manner. They showed an IC_50_ value of 155 μg/mL, and this potency increased to 266 μg/mL at 40 h. This indicates their potential as an anticancer agent for colon cancer treatment [106]. Another study investigated the cytotoxicity of Ag‐NPs derived from *Zingiber officinale* (ginger) rhizomes against HT‐29 colon cancer cells. Ag‐NPs exerted varying degrees of cytotoxicity, with an IC_50_ value of 150.8 µg/mL. This study indicated that higher concentrations showed a more pronounced cytotoxic effect [107]. In another study, the cytotoxicity of AgNO_3_ and Pectin/Ag NPs was assessed against normal (HUVEC) and colorectal carcinoma cell lines (HT‐29, HCT‐116, HCT‐8, and Ramos.2G6.4C10) using MTT assays over 48 h. The results demonstrated the dose‐dependent reduction in cell viability with increasing concentrations of AgNO_3_ and Pectin/Ag NPs across all cell lines. The IC_50_ values for Pectin/Ag NPs against the colorectal carcinoma cell lines were 485 µg/mL for Ramos.2G6.4C10 (a), 393 µg/mL for HCT‐8 [HRT‐18] (b), 236 µg/mL for HCT‐116 (c), and 262 µg/mL for HT‐29 (d) [108].

Studies have shown that *F. nucleatum,* a tumorigenic bacterium, promotes immunosuppressive *myeloid‐derived suppressor cells* that can hinder the host's anticancer immune response [109]. To prevent the proliferation of tumorigenic bacteria, the antibiotic therapy is commonly used. In recent years, research has explored the potential alternative strategies such as the utilization of silver ions application of silver ions to inhibit tumorigenic bacteria. The M13@Ag is assembled through electrostatic interaction and comprises inorganic AgNPs and the protein capsid of the bacteriophage M13. In an animal study, *F. nucleatum* was colonized in mice gut for 24 h, and after that M13@Ag was injected intravenously. The study demonstrated the specific binding affinity of M13@Ag toward *F. nucleatum* (Fn). This indicated making them promising candidates for targeted bacterial elimination. Additionally, the study showed antitumor effects of M13@Ag through a multifaceted approach including activation of antigen‐presenting cells, inhibition of myeloid‐derived suppressor cells, and suppression of regulatory T cells [110].

Silicon nanoparticles (SiNPs) possess a porous structure and offer advantageous characteristics such as pH sensitivity, biocompatibility, less toxicity, and ease of functionalization. These characteristics are recommended for precise drug delivery in anticancer applications. For instance, hybrid systems combining mesoporous SiNPs and protamine, as well as hyaluronic acid‐conjugated SiNPs, have been employed to enhance the effectiveness of drug delivery in the treatment of CRC. Additionally, SiNPs are used in combination with photosensitizers for targeted therapy, where photosensitizers become activated when exposed to light, releasing reactive oxygen molecules that can destroy cancer cells [111]. A recent study investigated the impact of Si NPs on GM after at dosages from 40 to 80 mg/kg body weight for 12 days. The study found an increased abundance of *Lactobacillus, Sphingomonas, Sutterella, Akkermansia,* and *Prevotella,* whereas Ruminococcus and Allobaculum decreased in abundance after exposure [83]. *Lactobacillus* showed the immunomodulatory property on Caco‐2 cells [112]. A recent study investigated the potential of P127‐MLL@Gin for modulating GM and its impact on colon cancer. P127‐MLL@Gin contained 6‐gingerol (Gin), mulberry leaf‐extracted lipids (MLLs), and Pluronic F127 (P127), which were loaded onto magnetic mesoporous silicon nanoparticles. The study demonstrated that oral administration of P127‐MLL@Gin, in combination with alternating magnetic fields, can activate antitumor immunity and suppress tumor growth in animal models. Additionally, the oral adminstartion of P127‐MLL@Gin resulted in the modulation of the GM composition specifically increased the abundance of *Bacillus* and unclassified c‐*Bacilli*, and reduced the *Bacteroides* and *Alloprevotella*. These findings suggest a potential interrelationship between P127 and MLL@Gin nanoparticles, GM modulation, and colon cancer development [113].

Copper sulfide NPs were incorporated into drug delivery systems for CRC treatment. These NPs acted synergistically with drugs like oxaliplatin, showing promise in treating CRC [114, 115]. Copper chelation may be used as a potential treatment strategy for cancers by altering the phosphorylation state of extracellular signal‐regulated kinase 1/2. Baldari et al. showed that copper chelation had an impact on colon cancer cells' ability to proliferate, survive, and migrate [116]. Table 2 represents the impact of MNPs on CRC in various models.

**Table 2:** Metallic nanoparticles (NPs) used for the treatment of colon cancer.

Metallic nanoparticles	Model	Outcomes	References
ZnO NPs	Caco‐2	Increased cell cycle arrest, cytotoxic activity, and median lethal dose were safer than *Croton tiglium* L. seed extract	[117]
ZnO NPs, Mo‐ZnO NPs, and Mo‐ZnO/RGO NCs	HCT‐116	Showed enhanced anticancer properties and improved cytocompatibility. The IC_50_ value of ZnO NPs is 45 μg/mL, Mo‐ZnO NPs is 30 μg/mL, and Mo‐ZnO/RGO NCs is 14 μg/mL	[118]
ZnO NPs	HCT‐116	Excellent cytotoxic effect in a dose‐dependent manner through reactive oxygen species generation	[119]
AgNPs from *Adansonia digitata* fruit extract	HCT‐116 and SW480	AgNPs were more cytotoxic than *Adansonia digitata* extract and decreased *LRP6* and *CTNNB1* gene expression	[120]
AuCl_4_/*Rosmarinus officinalis* leaves extract	HT‐29	Arrested HT‐29 cells at G2/M stages	[121]
Platinum nanoparticles (PtNPs)	HCT‐116 cells	Demonstrated antitumor properties through oxidative stress, apoptosis, and cytotoxicity	[122]
AgNPs	HCT‐116	Induced apoptosis via late‐stage apoptosis mechanism	[123]
AgNPs	HT‐29	Induced mitochondria‐dependent apoptosis and noncanonical autophagy	[124]
AgNPs	HCT‐116	Altered the metabolites secreted by gut microbes, such as increased folate levels, and reduced cancer cell viability	[125]
Bi‐doped cuprous oxide	CT26 cells	Showed excellent tumor targeting and photothermal therapeutic property	[126]
Black tea extract‐AuNP	HCT‐116	Cytotoxic effect involved apoptosis through a reactive oxygen species‐dependent pathway	[127]
AgNPs	HCT‐116	Demonstrated a cytotoxicity effect of 77.5%	[128]
Lentinan‐selenium nanoparticles (LNT‐SeNPs)	HCT‐116, HT‐29, Caco‐2, SW620, and CT26	Promoted apoptosis and induced cell cycle arrest at G0/G1 phase. The IC_50_ value is 7.65 μM	[129]
AgNP	Mouse CT26 colon carcinoma	Showed time‐ and concentration‐related antiproliferative effects	[130]
Fe_3_O_4_ NPs	Caco‐2	Demonstrated synergistic cytotoxicity with 5‐FU at low concentrations	[131]
CuNPs	SW480	Inhibited viability of cells, increased reactive oxygen species, and increased Bax and p53 gene expression	[132]
AgNPs were doped into bio‐based amorphous silica (Ag‐b‐SiO_2_)	HCT‐116	Size‐dependent cytotoxic activity and induced apoptosis	[133]
Ag‐NPs	Sw620 and HT‐29	Showed anticancer activity and biocompatibility against normal HFs cells	[134]
Silver–indium–sulfide quantum dots	HT29 and SW480 human colon adenocarcinoma cancer cell lines	Improved photodynamic therapy	[135]
Fe_3_O_4_@*White tea*/Agnanocomposite	HT‐29 and Caco‐2	The IC_50_ value observed in the HT‐29 cell lines was 384.2 μg/mL and in Caco‐2 was 254.6 μg/mL	[17]
*Arum dioscoridis*‐AgNPs	Caco‐2	The IC_50_ was determined as 2.977 μg/mL	[136]
AgNPs	HCT‐116	Did not induce cell death	[137]
AgNPs	NCM460 and Caco‐2	IC_50_ values for NCM460 and Caco‐2 cancer cells were 79.46 and 10.41 μg/mL, respectively	[138]
*Taraxacum officinale* leaves (TOL‐AgNPs)	Wistar albino rats	Decreased hyperplastic lesion Inhibited colon cancer induced by 1, 2‐dimethylhydrazine (DMH)	[139]
Gold‐silver core‐shell nanoparticles (Au‐AgNPs)	HCT‐116	PCA‐loaded Au‐AgNPs at a concentration of 15.63 μg/mL resulted in 80% inhibition of HCT‐116 cells	[140]
AgNPs	Caco‐2	Altered biological processes, cellular components, metabolic pathways, and hub gene expression contributing to the anticancer effects	[141]
Amorphous calcium phosphate nanoparticles (ACP NPs)	C57BL/6 mice	Reduction in tumor volume (62%) and a significant decrease in the number and size of polyps	[142]
AuNPs	Caco‐2	Dose‐dependent cytotoxic effect and IC_50_ is 21.31 ± 0.15 μg/mL	[143]
AgNPs	HCT‐116	Showed cytotoxic activity and IC_50_ is 29.5 μg/mL	[144]
MnNPs	HCT‐116 and SW480	Enhanced CPT efficacy against CRC. Significant reduction in cell viability after the treatment with 5‐FU‐loaded Mn‐based NPs as compared to free 5‐FU	[145]
Fe_3_O_4_ NPs	SW480 and SW620	Low cytotoxicity suitable for MRI‐guided hyperthermia	[146]
AuNPs	Human colorectal adenocarcinoma SW48	Synchrotron‐based X‐ray radiation and nanoparticles offer a targeted approach to treat cancer with minimal damage to healthy tissues	[147]
Honeybush‐AuNPs	Caco‐2	Cotreatment of HB‐AuNPs resulted in higher cell death	[148]
ZnO NPs	DLD‐1 (Human colon adenocarcinoma epithelial cell line)	Showed antiproliferative effects	[149]
CuO NPs	HCT‐116	IC_50_ is 25 µg/mL and exhibited cytotoxic effect	[150]
AgNPs	HT‐29	Induced apoptosis in a dose‐dependent manner	[107]
Fe_3_O_4_ NPs	CCD112 and HCT‐116	Showed anticancer activity possibly due to the release of iron ions from the Fe_3_O_4_ NPs	[98]
ZnO NPs	SW480	Cell viability decreased in a dose‐dependent manner and partly mediated by nitric oxide	[151]

## 6 CHALLENGES AND FUTURE DIRECTIONS

MNPs have both positive and negative impacts on the GM. However, these impacts were dependent on various factors such as MNP size, shape, surface charge, and composition; therefore, in‐depth investigation is still required. As stated above, humans are exposed to MNPs and the exposure depends on sources (food, cosmetics, and medications) and individual factors (diet and occupation). So, it is difficult to quantify the exact dosage and duration of MNP exposure in real‐world scenarios.

The challenge in using MNPs for CRC therapy is ensuring safety. Therefore, comprehensive studies are required for the assessment of toxicity and safety in the short and long term. As MNPs affect the GM, it is crucial to know the specific mechanisms and microbial responses to different MNPs and how these changes influence CRC. The majority of studies rely on in vitro models or animal studies. Translating these findings to humans needs well‐designed clinical trials to assess the effectiveness of NP‐based CRC therapies in the real world for validating the results for clinical utility. Precise targeting of MNPs is another challenge, for example, targeting CRC cells and sparing healthy cells. There is a need to develop MNPs that can specifically target and deliver therapeutic agents to CRC cells.

Exploring the synergistic effects of NPs with other approaches such as immunotherapy or conventional chemotherapy is required. Advancements in NP‐based imaging techniques can improve CRC diagnosis; thus, there is a need to explore the theranostics impact.

## 7 CONCLUSION

CRC is a global health concern and is projected to increase significantly by 2040, emphasizing the need for an effective approach. In CRC, there is a shift in GM that can impact gene expression and cancer progression. The MNPs (such as iron, zinc, and copper) show promise in CRC diagnosis and therapy. They can enhance drug delivery, enable targeted therapy, and may restore GM. However, the MNPs and GM interaction is complex and require further investigation.

## AUTHOR CONTRIBUTIONS

**Akash Kumar**: Data curation (lead); visualization (lead); writing—original draft (lead). **Jhilam Pramanik**: Visualization (equal); writing—original draft (equal). **Kajol Batta**: Writing—original draft (equal). **Pooja Bamal**: Writing—original draft (equal). **Mukesh Gaur**: Writing—original draft (equal). **Sarvesh Rustagi**: Writing—original draft (equal). **Bhupendra G. Prajapati**: Conceptualization (equal); supervision (lead); writing—review and editing (equal). **Sankha Bhattacharya**: Conceptualization (lead); supervision (lead); writing—review and editing (lead).

## CONFLICT OF INTEREST STATEMENT

The authors declare no conflict of interest.

## ETHICS STATEMENT

Not applicable.

## INFORMED CONSENT

Not applicable.

## Data Availability

Data sharing is not applicable to this article as no data sets were generated or analyzed during the current study.
